# Serum concentrations of medroxyprogesterone acetate were undetectable on OPU+5 days and had no effect on the serum progesterone level in patients undergoing the progestin-primed ovarian stimulation protocol

**DOI:** 10.3389/fendo.2025.1490839

**Published:** 2025-05-14

**Authors:** Xin Chen, Xu Yan, Hongyi Xu, Yueyue Hu, Shengfang Jiang, Xiaoning Wang, Haiying Peng, Bo Feng, Changjun Zhang, Honglu Diao, Ying Zhang

**Affiliations:** ^1^ Reproductive Medicine Center, Renmin Hospital, Hubei University of Medicine, Shiyan, China; ^2^ Hubei Clinical Research Center for Reproductive Medicine, Shiyan, China; ^3^ Biomedical Engineering College, Hubei University of Medicine, Shiyan, China; ^4^ Biomedical Research Institute, Hubei University of Medicine, Shiyan, China; ^5^ Hubei Key Laboratory of Embryonic Stem Cell Research, Hubei University of Medicine, Shiyan, China

**Keywords:** progestin-primed ovarian stimulation, medroxyprogesterone acetate, progesterone level, MPA concentrations, pregnancy outcome

## Abstract

**Objective:**

To evaluate the dynamics of serum medroxyprogesterone acetate (MPA) concentrations and their influence on serum progesterone (P) levels and pregnancy outcomes in the progestin-primed ovarian stimulation (PPOS) protocol. A total of 116 patients who underwent *in vitro* fertilization/intracytoplasmic sperm injection (IVF/ICSI) treatment using the PPOS protocol were included. Serum MPA levels were measured on the third, fifth, and seventh days of MPA use; on the day of human chorionic gonadotropin (hCG) trigger; and two and five days after oocyte pick-up (OPU).

**Results:**

The serum MPA concentration was 2.26 ± 2.11 nmol/L on the hCG trigger day, 0.37 ± 0.40 nmol/L two days after OPU, and zero five days after OPU. There were no statistically significant differences in P levels on the hCG trigger day, total dosage of Gn, duration of Gn, number of oocytes retrieved, number of mature oocytes, fertilization rate, blastocyst progression rate, CPR, ectopic pregnancy rate, early pregnancy loss rate, or live birth rate (LBR) between the two cohorts (*P* > 0.05).

**Conclusion(s):**

Serum concentrations of MPA had no effect on serum P levels or pregnancy outcomes in patients undergoing the PPOS protocol.

## Introduction

In recent years, the progestin-primed ovarian stimulation (PPOS) protocol, which uses oral progestins as a substitute for gonadotropin-releasing hormone (GnRH) analogs to suppress the pituitary gland and inhibit premature luteinizing hormone (LH) surges, has emerged as an alternative to conventional protocols such as the GnRH agonist (GnRH-a) long protocol and antagonist protocol. The PPOS protocol has been applied in cases of oocyte donation (1, 2), fertility preservation (3, 4), and hyper-responders (5). Its advantages include lower gonadotropin consumption (6), the retrieval of more oocytes and good embryos, and a higher cumulative live birth rate (CLBR) in older women (7) and women with diminished ovarian reserve (DOR) (8), as well as comparable pregnancy outcomes in women with normal ovarian reserve (9). Consequently, the PPOS protocol is considered more convenient, effective, and suitable for all *in vitro* fertilization/intracytoplasmic sperm injection (IVF/ICSI) patients (6, 10, 11).

Since oral progestins are analogs of progesterone (P), exposure to these drugs throughout the body may impact three stages: the oocyte developmental stage during ovulation induction, the embryo implantation stage, and the early developmental stage of embryos. This exposure could lead to lower oocyte quality, reduced endometrial receptivity (12) and embryonic teratogenicity and toxicity (13). Therefore, a freeze-all and thawed embryo transfer (ET) strategy is routinely implemented in the PPOS protocol.

The grade rating of embryos, the aneuploidy rate, and the clinical pregnancy rate (CPR) can indirectly reflect oocyte quality. Previous studies have shown that the number of blastocysts, euploid blastocyst rates, and CPRs were similar between PPOS patients and those undergoing conventional stimulation cycles (6, 10, 11, 14–19). Additionally, there are concerns regarding the potential risks of progestins on the health and safety of offspring in patients undergoing the PPOS protocol. Some studies have reported no significant differences in neonatal outcomes or congenital malformation rates between the PPOS protocol and traditional protocols (20–24). Moreover, oral progestins are assumed to increase progesterone (P) levels, particularly on the human chorionic gonadotropin (hCG) trigger day, potentially shifting the endometrial implantation window. However, this theory remains controversial (17, 25–38).

The PPOS protocol has been tested with various progestin administration methods, with medroxyprogesterone acetate (MPA) being the most commonly used. Evaluating the potential influence of the MPA dose effect is crucial. Our previous study demonstrated that a degressive administration of MPA based on serum luteinizing hormone (LH) levels could decrease the total MPA dose while preventing preovulation (39). Therefore, it is important to understand the duration of MPA metabolism in the PPOS protocol and assess whether different serum MPA concentrations affect P levels and pregnancy outcomes. We conducted a single-center retrospective cohort study to explore the dynamics of serum MPA levels and to compare P levels on the hCG trigger day and pregnancy outcomes after frozen embryo transfer (FET) between high- and low-MPA groups, which is clinically significant.

## Materials and methods

### Study design and patients

We conducted a hospital-based retrospective cohort study, adhering to the principles outlined in the Declaration of Helsinki. Data were collected from the Reproductive Medicine Center, Renmin Hospital, Hubei University of Medicine, covering the period from October 2021 to October 2022. All the data were anonymized to ensure patient confidentiality and privacy.

Women who underwent the PPOS protocol were included in the study if they met the following criteria: patients with regular menstrual cycles (25-35 days), aged 20-40 years, body mass index (BMI) 18-28 kg/m², bilateral antral follicle counts (AFCs) 3-20, and normal basal serum levels of follicle-stimulating hormone (FSH) (<10 IU/L) and anti-Müllerian hormone (AMH) (≥1.1 ng/mL) on Day 2 or 3 of the cycle before ovarian stimulation. The exclusion criteria included metabolic disorders, polycystic ovarian syndrome (PCOS), endometriosis, pelvic tuberculosis, congenital uterine malformations, chromosomal abnormalities, single-gene disorders, and immunological diseases.

The ovarian stimulation was started on Day 2 or 3 of the cycle. The detailed treatment of a modified PPOS protocol and endometrial preparation methods for FET used in this study has been reported in our previous research (39).

Moderate/severe OHSS was diagnosed in women who met more than one of the following criteria: clinical ascites, hydrothorax, or dyspnea (exertional or at rest). Biochemical pregnancy was defined as hCG >10 IU/L two weeks after ET. Clinical pregnancy was defined as an intrauterine gestational sac identified by ultrasonography 30 days after ET. Early pregnancy loss was defined as spontaneous pregnancy loss before 12 weeks. Live birth was defined as a living fetus born after 28 weeks of pregnancy.

### Outcome parameters

Serum FSH, LH, E_2_, and P levels were measured on the first day of stimulation; the third, fifth, and seventh days of MPA use; and the hCG trigger day. Hormone levels were determined using electrochemiluminescence (Beckman Coulter, USA), with all measurements conducted by skilled technicians in accordance with the manufacturer’s instructions. The detection sensitivity limits were as follows: FSH, 0.2 IU/L; LH, 0.2 IU/L; E_2_, 15 pg/ml; and P, 0.1 ng/ml. The inter- and intra-assay coefficients of variation were less than 10%.

Serum MPA levels were measured on the third, fifth, and seventh days of MPA use; on the hCG trigger day; and two and five days after OPU. MPA was extracted from 1,000 μl of serum and evaporated to dryness, and the reconstituted solution was injected onto a Waters Acquity liquid chromatography (LC) system using an Agilent Zorbax Eclipse-Plus C18 2.1 × 100 mm (3.0 μm) column. MPA and its internal standard were monitored on a QTRAP^®^ 5500 mass analyzer in positive ionization mode. The method was validated according to the FDA Bioanalytical Method Validation guidelines. The calibration curve for MPA had a linear range of 0.10-8.0 μg/l, with a limit of quantification of 40 ng/l. The relative recovery was 76.0%. The inter- and intraday precisions were less than 9.0%.

### Statistical methods

All analyses were performed using EmpowerStats (http://www.empowerstats.com) and SPSS 26.0 (IBM, Armonk, NY, USA). Continuous variables are presented as the means with standard deviations or medians with interquartile ranges, and differences among groups were compared using one-way analysis of variance or the Kruskal–Wallis test. A multivariable regression model was constructed to identify factors related to pregnancy outcomes in all participants. Statistical significance was set at a two-sided *P* value < 0.05. Graphs were created from histograms constructed with GraphPad Prism version 8.4.2 (GraphPad, La Jolla, CA).

### Ethics statement

The study protocol was reviewed and approved by the Ethics Committee of Renmin Hospital, Hubei University of Medicine (No: SYRMYY-065). Informed consent was obtained from all patients at the time of enrollment.

## Results

In our retrospective cohort study, we achieved a total of 116 OPU cycles and 97 FET cycles. In the remaining 19 patients, no embryos were available for transfer.

### Dynamics of serum MPA concentrations

The total dosage of MPA was 21.06 ± 10.38 mg, and the duration of MPA administration was 6.21 ± 1.94 days. The serum MPA concentrations were as follows: 4.27 ± 1.09 nmol/L on the third day, 4.86 ± 1.97 nmol/L on the fifth day, 4.35 ± 2.85 nmol/L on the seventh day, 2.26 ± 2.11 nmol/L on the hCG trigger day, 0.37 ± 0.40 nmol/L two days after OPU, and zero five days after OPU (**Supplementary Table 1**).

**Figure 1:**
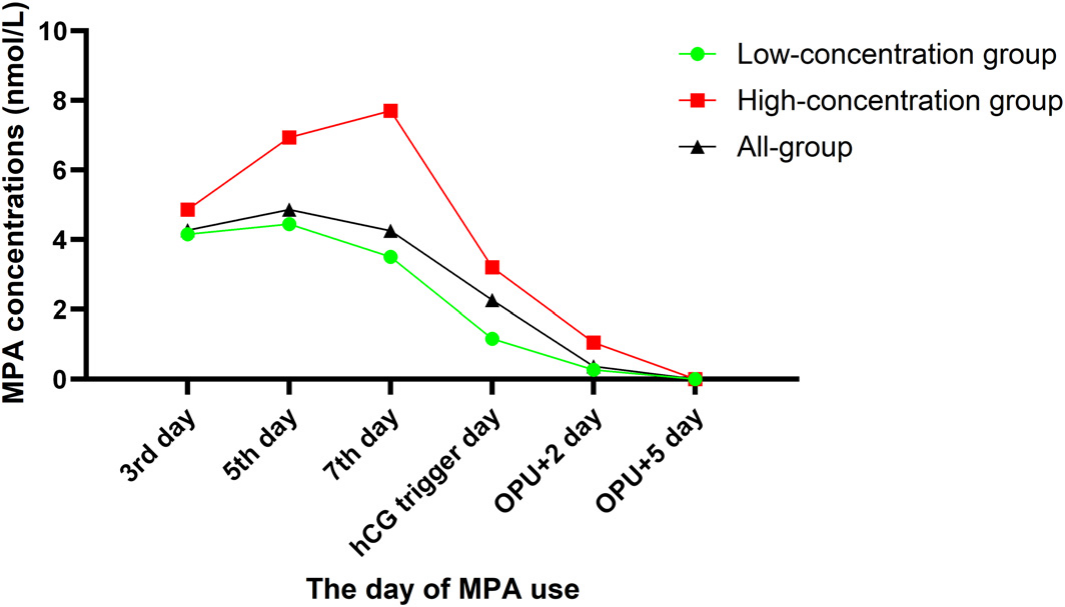
Different groups of the serum MPA concentrations after administration of MPA in the PPOS protocol.

All women were divided into two groups according to a cutoff value of 2.5 nmol/L for serum MPA concentrations on the hCG trigger day. The serum MPA concentrations at any time point were higher in the high-concentration group than that in the low-concentration group, expect on the OPU+5 day (**Figure 1**).

### Data on ovulation induction process and embryological outcomes

The patient characteristics of the two groups are provided in **Table 1**. Significant differences were observed in the MPA dose, MPA duration, serum MPA concentration, and type of infertility (*P* < 0.05). However, there were no statistically significant differences between the two groups in terms of female age, BMI, AMH, AFC, duration of infertility, or insemination method (*P* > 0.05).

**Table 1:** Baseline characteristics of women with different serum MPA concentrations on the hCG trigger day in the PPOS protocol.

	Low concentration group	High concentration group	*P* value
No. of cycles	42	74	/
MPA dose (mg)	16.17± 8.42	24.26 ± 7.38	0.014
MPA duration (days)	4.21 ± 1.97	6.38 ± 1.65	0.048
Plasma MPA concentrations (nmol/l)	2.14 ± 1.17	3.34 ± 1.89	0.021
Female Age (years)	30.54 ± 3.83	32.48 ± 5.14	0.321
BMI (kg/m^2^)	23.47 ± 3.36	24.47 ± 2.05	0.764
AMH (ng/ml)	2.34 ± 2.09	2.76 ± 2.23	0.873
AFC	6.63 ± 3.26	7.21 ± 3.82	0.123
Duration of infertility (years)	3.91 ± 3.35	3.12 ± 2.26	0.189
Infertile patients, n (%)			0.019
Primary infertility	12 (28.57%)	28 (37.83%)	
Secondary infertility	30 (71.43%)	46 (62.17%)	
Insemination method			0.250
IVF	29 (69.05%)	55 (74.32%)	
ICSI	13 (30.95%)	19 (26.38%)	

Date: mean ± SD or (%) (no./total no.). PPOS, progestin-primed ovarian stimulation; MPA, medroxyprogesterone acetate; BMI, body mass index; AMH, anti-Mullerian hormone; AFC, antral follicle count; IVF, *in vitro* fertilization; ICSI, intracytoplasmic sperm injection.

The ovarian stimulation characteristics of the two groups are summarized in **Supplementary Table 2**. No statistically significant differences were found between the two groups regarding the total dosage of Gn, duration of Gn, number of oocytes retrieved, number of mature oocytes, fertilization rate, blastocyst progression rate, number of frozen embryos, or moderate/severe OHSS rate (*P* > 0.05).

### Hormone profile data

**Figure 2:**
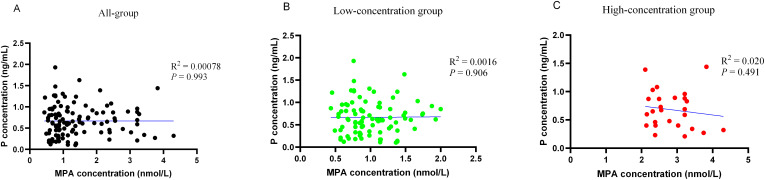
Scatterplots and correlations (Pearson correlation coefficients) of women with different serum MPA concentrations and P concentrations on the hCG trigger day. **(A)** All the serum MPA concentration group. **(B)** Low serum MPA concentration group. **(C)** High serum MPA concentration group.

During ovarian stimulation, there were no statistically significant differences in LH, E_2_, or P levels between the two cohorts at any time point (*P* > 0.05) (**Supplementary Table 3**). Linear regression revealed no correlation between serum MPA concentration and P concentration on the hCG trigger day, with correlation coefficient R^2^ = 0.00078, *P* = 0.993 (**Figure 2**).

### Clinical outcome data

Descriptive statistics for the reproductive outcomes of FET are summarized in **Table 2**. There were no statistically significant differences between the two groups in terms of endometrial preparation method, number of transferred embryos, embryo transfer stage, CPR, ectopic pregnancy rate, early pregnancy loss rate, or live birth rate (LBR) (*P* > 0.05).

**Table 2:** Reproductive outcomes of freeze–thaw transplantation cycles in patients with different serum MPA concentrations in the PPOS protocol.

	Low concentration group	High concentration group	*P* value
No. of cycles	35	62	
Endometrium preparation protocol			0.897
Down regulation +HRT	26 (74.28%)	47 (75.80%)	
HRT	9 (25.72%)	15 (24.20%)	
No. of transferred embryos, n (%)			0.914
One	23 (65.71%)	42 (67.74%)	
two	12 (34.29%)	20 (32.26%)	
Embryo transfer day, n (%)			0.621
Day 3	8 (22.86%)	12 (19.35%)	
Day 5	27 (77.14%)	50 (80.65%)	
Implantation rate (%)	76.60 (36/47)	73.17 (60/82)	0.453
Biochemical pregnancy rate (%)	57.14 (20/35)	64.51 (40/62)	0.238
Clinical pregnancy rate (%)	48.57 (17/35)	51.61 (32/62)	0.425
Ectopic pregnancy rate (%)	5.88 (1/17)	3.13 (1/32)	0.628
Early pregnancy loss rate (%)	11.76 (2/17)	15.63 (4/32)	0.524
Live birth rate (%)	42.86(15/35)	43.54(27/62)	0.763
No. of fetuses in pregnancy, n (%)			0.752
single	11 (73.33%)	20 (74.07)	
twins	4 (26.67%)	7 (25.93)	

MPA, medroxyprogesterone acetate; PPOS, progestin-primed ovarian stimulation; HRT, hormone replacement therapy.

Multiple regression analysis indicated that variations in the total MPA dosage were not significantly related to changes in hormone levels on the hCG trigger day, CPR, or LBR in either the unadjusted or adjusted models.

## Discussion

To our knowledge, this is the first report on serum MPA dynamics in patients undergoing the PPOS protocol during IVF/ICSI treatment. We observed a decline in MPA levels during the late follicular stage following a gradual reduction in MPA dosage, with levels becoming undetectable by the fifth day after OPU, coinciding with blastocyst transfer, potentially allowing for fresh ET in the PPOS protocol. Furthermore, both the high- and low-MPA groups presented comparable P levels on the hCG trigger day, suggesting that MPA administration in the PPOS protocol does not affect endometrial receptivity.

MPA has historically been used as a contraceptive agent. As a potent synthetic progestin, it exhibits a distinct metabolism compared with that of P due to structural differences. Previous studies have shown that following oral administration of a single dose of MPA, serum levels peak within 1 to 4 hours and then decline rapidly, with a biological half-life of 40-60 hours (25, 40, 41). In our study, MPA was administered from Day 5 of Gn use until the hCG trigger day, resulting in serum levels of 2.26 ± 2.11 nmol/L on the hCG trigger day, 0.37 ± 0.40 nmol/L on OPU+2 day, and undetectable levels on OPU+5 days. Consistent with the literature, our findings indicate rapid clearance of MPA in women undergoing the PPOS protocol. As an analog of P, MPA may exert similar effects. When serum MPA levels decrease to undetectable levels, the P-like activity diminishes, potentially facilitating fresh ET at the blastocyst stage (OPU+5 days) rather than the cleavage stage (OPU+3 days).

Endometrial receptivity can be assessed through morphological observation or biomarker profiling of endometrial function. In a randomized controlled trial (RCT), MPA induced a significant increase in subnuclear vacuolation, a classical effect of P, at oral doses of 2.5 mg, 5 mg, and 10 mg per day during the mid-proliferative stage over 4 days (42). Another study demonstrated that, unlike P, MPA promoted the differentiation of human monocytes toward an M2 phenotype, resembling decidual macrophages that are crucial for successful pregnancy, via extracellular regulated protein kinase (ERK) phosphorylation in both a human monocyte cell line and primary monocytes (43). Furthermore, a transcriptome and biofunctional study using primary human stromal cell cultures revealed the differential expression of 116 genes with P treatment and 251 genes with MPA treatment compared with the vehicle control (44). Both treatments upregulated genes such as SPARCL1, SLC7A8, OMD, FKBP5, THSD7A, LCP1, GPX3, and IL1R1, while downregulating EVT1, NDNF, LYPD1, GBP4, KRT19, SFRP1, and CD34. Notably, both treatments decreased cell viability. Therefore, further investigations are needed to clarify the impact of MPA administration on endometrial receptivity and elucidate the underlying mechanisms, thereby offering insights into the use of MPA during ovulation induction.

The detrimental effects of premature elevation of P on the hCG trigger day during IVF/ICSI treatment have been extensively documented (45). Hence, attention should be focused on serum P levels potentially altered after progestin administration. A study involving women treated by MPA for threatened abortion in the first trimester reported no difference in urine P levels between treated and untreated women (46). The serum P levels remained consistent with the follicular phase levels during and up to 20 days after treatment with intravaginal administration of a single 100 mg dose for 21 days (25). In poor responders, P levels remained low at the LH surge day in natural cycles but were higher in the minimal stimulation MPA group (26). In a comparison of the serum P levels on the hCG trigger day among patients who underwent IVF/ICSI cycles using 4 mg PPOS or short-term protocols, no significant difference was found between the 4 mg PPOS protocol and the short-term protocol, but the P levels were greater in the 10 mg PPOS protocol (27), although contradictory results have been reported (33). Most studies, including self-controlled studies and RCTs, have consistently shown comparable serum P levels on the hCG trigger day between the PPOS group and traditional protocol groups (17, 28–32, 34–37, 39, 47, 48). Similarly, our study revealed no difference in P concentration on the day of ovulation trigger between the high- and low-MPA groups, as defined by serum MPA levels on the hCG trigger day, suggesting that MPA levels do not impact P secretion.

Evidence regarding the safety of MPA use during the first trimester remains limited. A large population study involving 1,016 women revealed no significant difference in congenital abnormalities between women treated orally with MPA at doses of 80–120 mg per day for at least three months (4.1%, 15/366 infants) and those in an untreated group (3.5%, 15/428 infants) (49). Similar results were observed in a female baboon experiment (50). However, clinical reviews have reported male feminization and female masculinization (51), and experiments on cynomolgus monkeys have revealed female pseudohermaphroditism, male hypospadias, and reduced adrenal gland size (52). Our study used a low dosage of 10 mg MPA, which was significantly lower than that used in previous studies. Thus, we hypothesize that at such low doses, MPA may be safe for early embryonic development without embryotoxic or teratogenic effects.

Our study has several limitations. First, the lack of data on blastocyst euploidy rates may impact the precision of our conclusions. Second, we did not measure corresponding P levels at the timepoints of MPA measurement, limiting our understanding of P dynamics following MPA use. Third, molecular-level results from human endometrial tissues were absent. Fourth, our sample size was relatively limited and no formal power analysis was performed, as this was an exploratory retrospective study. Fifth, the patient cohort was heterogeneous and not stratified by PGT-A or oocyte donor cycles, which may reduce the generalizability of our findings. Lastly, as our study focused solely on MPA, extrapolating these results to other progestins should be done with caution.

## Conclusion

This retrospective study suggests that serum MPA levels were undetectable on the fifth day after OPU and that serum P levels were unaffected by MPA levels on the hCG trigger day. While these observations may indicate the potential for fresh blastocyst transfer in the PPOS protocol, the findings should be interpreted cautiously given the retrospective nature, modest sample size, and lack of mechanistic confirmation. Further prospective studies are needed to validate the clinical applicability of these findings and their implications for treatment strategy optimization.

## Data Availability

The data analyzed in this study is subject to the following licenses/restrictions: The raw data supporting the conclusions of this article will be made available by the authors, without undue reservation. Requests to access these datasets should be directed to yingzhangivf@gmail.com.
