# Supplementary material for: Serum concentrations of medroxyprogesterone acetate were undetectable on OPU+5 days and had no effect on the serum progesterone level in patients undergoing the progestin-primed ovarian stimulation protocol
Source: Front Endocrinol (Lausanne). 2025 May 14;16:1490839. doi: 10.3389/fendo.2025.1490839 (PMC12116319; doi:10.3389/fendo.2025.1490839)
Supplement: Supplementary file 1 [file Table1.docx]

**Supplemental Table 1** Plasma MPA concentrations after MPA administration

| Variables | Value |
| --- | --- |
| No. of cycles | 116 |
| Dosage of MPA (mg) | 21.06 ± 10.38 |
| Duration of MPA (days) | 6.21 ± 1.94 |
| Plasma MPA concentrations (nmol/l) |  |
| on 3rd day of MPA use (day 7 of Gn use) | 4.27 ± 1.09 |
| on 5th day of MPA use (day 9 of Gn use) | 4.86 ± 1.97 |
| on 7th day of MPA use (day 11 of Gn use) | 4.35 ± 2.85 |
| on hCG trigger day | 2.26 ± 2.11 |
| on OPU+2 day | 0.37 ± 0.40 |
| on OPU+5 day | 0.00 |

Date: mean ± SD or (%) (no./total no.). MPA, medroxyprogesterone acetate; hCG, human chorionic gonadotropin; OPU, oocyte pick-up.
