# Supplementary material for: Serum concentrations of medroxyprogesterone acetate were undetectable on OPU+5 days and had no effect on the serum progesterone level in patients undergoing the progestin-primed ovarian stimulation protocol
Source: Front Endocrinol (Lausanne). 2025 May 14;16:1490839. doi: 10.3389/fendo.2025.1490839 (PMC12116319; doi:10.3389/fendo.2025.1490839)
Supplement: Supplementary file 2 [file Table2.docx]

**Supplemental Table 2** Ovarian stimulation characteristics and embryological outcomes of women with different serum MPA concentrations on the hCG trigger day in the PPOS protocol

|  | Low concentration group | High concentration group | *P* value |
| --- | --- | --- | --- |
| Dosage of Gn (IU) | 1977.19 ± 542.11 | 2208.32 ± 688.02 | 0.05 |
| Duration of Gn (days) | 10.72± 1.55 | 11.88 ± 1.34 | 0.211 |
| No. of oocytes retrieved | 6.23 ± 3.10 | 6.24 ± 2.50 | 0.889 |
| No. of mature oocytes | 5.49 ± 3.22 | 5.16 ± 2.68 | 0.775 |
| Fertilization rate (2PN) (%) | 93.77 (301/321) | 94.25 (525/557) | 0.867 |
| Cleavage rate (%) | 99.48 (577/580) | 99.74 (778/780) | 0.998 |
| No. of embryos obtained | 2.97 ± 1.88 | 3.02 ± 1.69 | 0.456 |
| No. of top-quality embryos | 1.84 ± 1.34 | 1.98 ± 1.60 | 0.332 |
| Blastocyst progression rate (%) | 64.40 (76/118) | 63.04 (145/230) | 0.426 |
| No. of frozen embryos | 1.91 ± 1.52 | 1.82± 1.70 | 0.732 |
| Moderate or severe OHSS, n (%) | / | / | / |

Date: mean ± SD or (%) (no./total no.). MPA, medroxyprogesterone acetate; hCG, human chorionic gonadotropin; PPOS, progestin-primed ovarian stimulation; Gn, gonadotropin; PN, pronuclear number; OHSS, ovarian hyperstimulation syndrome.
