# Supplementary material for: Serum concentrations of medroxyprogesterone acetate were undetectable on OPU+5 days and had no effect on the serum progesterone level in patients undergoing the progestin-primed ovarian stimulation protocol
Source: Front Endocrinol (Lausanne). 2025 May 14;16:1490839. doi: 10.3389/fendo.2025.1490839 (PMC12116319; doi:10.3389/fendo.2025.1490839)
Supplement: Supplementary file 3 [file Table3.docx]

**Supplemental Table 3** Hormone profiles of women with different serum MPA concentrations on the hCG trigger day in the PPOS protocol

|  | Low concentration group | High concentration group | *P* value |
| --- | --- | --- | --- |
| Hormones on 1st day of stimulation |  |  |  |
| LH (IU/L) | 3.18± 2.47 | 2.87± 2.12 | 0.623 |
| E_2_ (pg/ml) | 37.86± 17.68 | 31.54 ± 21.23 | 0.214 |
| P (ng/ml) | 0.67± 0.34 | 0.48 ± 0.27 | 0.553 |
| Hormones on 1st day of MPA use |  |  |  |
| LH (IU/L) | 3.87± 1.68 | 3.36± 2.13 | 0.098 |
| E_2_ (pg/ml) | 99.04±31.39 | 116.53± 65.42 | 0.143 |
| Hormones on 3rd day of MPA use |  |  |  |
| LH (IU/L) | 4.72± 2.43 | 5.08± 3.10 | 0.563 |
| E_2_ (pg/ml) | 336.87±153.49 | 387.96±163.98 | 0.458 |
| Hormones on 5th day of MPA use |  |  |  |
| LH (IU/L) | 4.98± 2.19 | 5.26± 1.89 | 0.543 |
| E_2_ (pg/ml) | 764.63±231.62 | 874.98± 324.82 | 0.198 |
| Hormones on 7th day of MPA use |  |  |  |
| LH (IU/L) | 4,35± 1.68 | 4.77± 2.13 | 0.673 |
| E_2_ (pg/ml) | 1006.65± 322.18 | 1265.76± 423.43 | 0.376 |
| Hormones on hCG trigger day |  |  |  |
| LH (IU/L) | 3.66± 2.23 | 3.35± 1.87 | 0.453 |
| E_2_ (pg/ml) | 1554.36± 532.31 | 1875.39± 624.39 | 0.412 |
| P (ng/ml) | 0.98± 0.63 | 0.82± 0.47 | 0.889 |

Date: mean ± SD or (%) (no./total no.). MPA, medroxyprogesterone acetate; hCG, human chorionic gonadotropin; PPOS, progestin-primed ovarian stimulation; LH, luteinizing hormone; E_2_, estradiol; P, progesterone.
