# Supplementary material for: Serum concentrations of medroxyprogesterone acetate were undetectable on OPU+5 days and had no effect on the serum progesterone level in patients undergoing the progestin-primed ovarian stimulation protocol
Source: Front Endocrinol (Lausanne). 2025 May 14;16:1490839. doi: 10.3389/fendo.2025.1490839 (PMC12116319; doi:10.3389/fendo.2025.1490839)
Supplement: Supplementary file 4 [file Table4.docx]

| **Supplemental Table 4** Multivariate logistic regression analysis of the effect of total MPA dosage on clinical pregnancy rate | | | | |
| --- | --- | --- | --- | --- |
| Factor | Model 1 | | Model 2 | |
|  | *OR(95% CI)* | *P value* | *OR(95% CI)* | *P value* |
| Endometrium preparation protocol | 0.999(0.989~1.009) | 0.998 | 1.032(0.958~1.112) | 0.403 |
| No. of transferred embryos | 1.47 (1.28~1.70) | <0.001 | 1.53 (1.31~1.78) | <0.001 |
| Embryo transfer day | 1.59 (1.12~2.26) | 0.009 | 1.63 (1.15~2.31) | 0.006 |
| Total MPA dose | 0.974(0.878~1.081) | 0.621 | 1.137(0.836~1.547) | 0.413 |
| serum MPA concentrations on the hCG trigger day | 1.207(1.008~1.445) | 0.121 | 1.132(0.759~1.686) | 0.467 |

Date: Model 1: Unadjusted model without controlling for confounding factors. Model 2: Adjusted for female age, duration of infertility, body mass index (BMI), anti-Müllerian hormone (AMH) level, antral follicle count (AFC), duration of stimulation, and total gonadotropin dose. OR: Odds ratio; CI: Confidence interval.
